# Optical Coherence Tomography Angiography in Type 1 Diabetes Mellitus—Report 2: Diabetic Kidney Disease

**DOI:** 10.3390/jcm11010197

**Published:** 2021-12-30

**Authors:** Aníbal Alé-Chilet, Carolina Bernal-Morales, Marina Barraso, Teresa Hernández, Cristian Oliva, Irene Vinagre, Emilio Ortega, Marc Figueras-Roca, Anna Sala-Puigdollers, Cristina Esquinas, Marga Gimenez, Enric Esmatjes, Alfredo Adán, Javier Zarranz-Ventura

**Affiliations:** 1 Institut Clínic d’Oftalmologia (ICOF), Hospital Clínic, 08028 Barcelona, Spain; anibalale@gmail.com (A.A.-C.); carolbernalmo@gmail.com (C.B.-M.); marinabarraso@gmail.com (M.B.); tessa.hrndz@gmail.com (T.H.); cristianolpa10@gmail.com (C.O.); mafiguer@clinic.cat (M.F.-R.); ansala@clinic.cat (A.S.-P.); amadan@clinic.cat (A.A.); 2 August Pi i Sunyer Biomedical Research Institute (IDIBAPS), 08036 Barcelona, Spain; ivinagre@clinic.cat (I.V.); eortega1@clinic.cat (E.O.); gimenez@clinic.cat (M.G.); esmatjes@clinic.cat (E.E.); 3 Medical Retina Service, Moorfields Eye Hospital NHS Foundation Trust, London EC1V 2PD, UK; 4 Diabetes Unit, Hospital Clínic, 08036 Barcelona, Spain; 5 Institut Clínic de Malalties Digestives i Metabòliques (ICMDM), Hospital Clínic, 08036 Barcelona, Spain; 6 Centro de Investigación Biomédica en Red de la Fisiopatología de la Obesidad y Nutrición (CIBEROBN), 08036 Barcelona, Spain; 7 Respiratory Department, Hospital Universitari Vall d’Hebron, 08035 Barcelona, Spain; crise4@hotmail.com

**Keywords:** diabetic nephropathy, oculomics, vessel density, perfusion density, foveal avascular zone, optical coherence tomography angiography, diabetic kidney disease

## Abstract

The purpose of this study is to investigate potential associations between optical coherence tomography angiography (OCTA) parameters and diabetic kidney disease (DKD) categories in type 1 diabetes mellitus (T1DM) patients and controls. A complete ocular and systemic examination, including OCTA imaging tests and bloods, was performed. OCTA parameters included vessel density (VD), perfusion density (PD), foveal avascular zone area (FAZa), perimeter (FAZp) and circularity (FAZc) in the superficial vascular plexus, and DKD categories were defined according to glomerular filtration rate (GFR), albumin-creatinine ratio (ACR) and KDIGO prognosis risk classifications. A total of 425 individuals (1 eye/1 patient) were included. Reduced VD and FAZc were associated with greater categories of GFR (*p* = 0.002, *p* = 0.04), ACR (*p* = 0.003, *p* = 0.005) and KDIGO risk prognosis classifications (*p* = 0.002, *p* = 0.005). FAZc was significantly reduced in greater KDIGO prognosis risk categories (low risk vs. moderate risk, 0.65 ± 0.09 vs. 0.60 ± 0.07, *p* < 0.05). VD and FAZc presented the best diagnostic performance in ROCs. In conclusion, OCTA parameters, such as VD and FAZc, are able to detect different GFR, ACR, and KDIGO categories in T1DM patients and controls in a non-invasive, objective quantitative way. FAZc is able to discriminate within T1DM patients those with greater DKD categories and greater risk of DKD progression.

## 1. Introduction

Diabetes mellitus (DM) is a systemic disease which affects the microvasculature of multiple organs leading to diabetic retinopathy (DR), nephropathy and neuropathy, with high impact on the quality of life of diabetic patients [1]. DR is caused by the alteration of the retinal capillary flow, producing retinal ischemia and, in later stages, neovascularization, being responsible for 2.6% of worldwide blindness in 2010 [2]. Diabetic nephropathy, also termed as diabetic kidney disease (DKD), usually develops 5 years after Type 1 diabetes mellitus (T1DM) diagnosis and occurs in 20–40% of DM patients, being currently the worldwide leading cause of chronic kidney disease (CKD) [3]. In T1DM patients, an association between DR and DKD has been demonstrated in the DCCT/EDIC study, which showed that DR progression and development of DKD were directly related, supporting the fact that both share an etiologic basis and present a common pathophysiology [4,5].

Optical coherence tomography angiography (OCTA) [6] is a newly developed, non-invasive, retinal imaging technique that allows objective quantification of microvascular parameters in the perifoveal vascular network, such as vessel density or flow impairment areas [7,8]. Recent studies have analyzed the relationship between OCTA and DR stage [7,9,10,11,12,13,14,15,16,17,18,19,20,21,22,23] and systemic markers of disease, such as glycated hemoglobin levels [24]. Since this technique allows direct noninvasive in vivo visualization of the microvascular circulation, it is sensible to think that the detection of microvascular changes may be associated to other clinical manifestations elsewhere in the body. With this concept, the relationship between OCTA and kidney disease has been investigated in recent reports that have described existing relationships between OCTA parameters and non-specific CKD [25,26] and DKD [13,27,28,29,30]. However, the vast majority of these studies have not been specifically directed to investigate the diagnostic potential of OCTA parameters compared to standard kidney function tests. Most have been conducted in relatively small series of patients and have predominantly been performed in type 2 DM patients.

The aim of this report is to specifically evaluate the association between OCTA parameters and DKD stages in different classifications in a large cohort of T1DM patients and controls. The clinical relevance of establishing these associations relies on the potential ability of OCTA to estimate kidney damage stages with a non-invasive eye imaging technique. Thus, it may allow clinicians to classify patients according to their risk and prognosis and act consequently, concentrating health resources in those patients at higher risk of developing microvascular complications.

## 2. Materials and Methods

### 2.1. Study Design and Protocol

Cross-sectional, exploratory study in a large cohort of T1DM patients with prospective collection of OCTA images and ocular and systemic clinical data. The study protocol has been described elsewhere [31]. This project was approved by the Institutional Review Board at the Hospital Clinic of Barcelona (HCB/2016/0216, study protocol v0.2), and it is registered in the Clinical Trials website (ClinicalTrials.gov, accessed on 6 February 2018, NCT03422965). Written informed consent was obtained for all participants.

### 2.2. Inclusion and Exclusion Criteria

T1DM patients undergoing routine follow-up visits at the Diabetes Unit were invited to participate and referred to the Ophthalmology department for a comprehensive ocular examination. Healthy controls were also recruited from social media campaigns supported by the Communications department of the Hospital. Exclusion criteria included ocular media opacities, ocular comorbidities (i.e., macular edema, previous ocular surgery, macular laser, intravitreal therapies, glaucoma, amblyopia, myopia, retinal vein occlusions and uveitis) and inability to perform complete ocular examinations or provide written informed consent. For this specific report, an additional inclusion criteria was the existence of kidney function tests performed within 1 month of the ocular examination.

### 2.3. Ocular and Systemic Data

The complete details of the ocular examination and systemic status assessment of diabetes has been described elsewhere [31]. Ocular data included best-corrected visual acuity (BCVA), biomicroscopy, intraocular pressure, fundus exam and biometry (IOL Master, Carl Zeiss Meditec, Dublin, CA, USA). DR stage was graded using the International Scale [32]. A comprehensive battery of OCT and OCTA images was performed as described below. Systemic data collected included age, sex, smoking habit, blood pressure, blood hypertension, body mass index and DM-related characteristics (i.e., DM duration, macrovascular complications, insulin requirements, etc.).

### 2.4. OCTA Imaging Protocol

**Figure 1:**
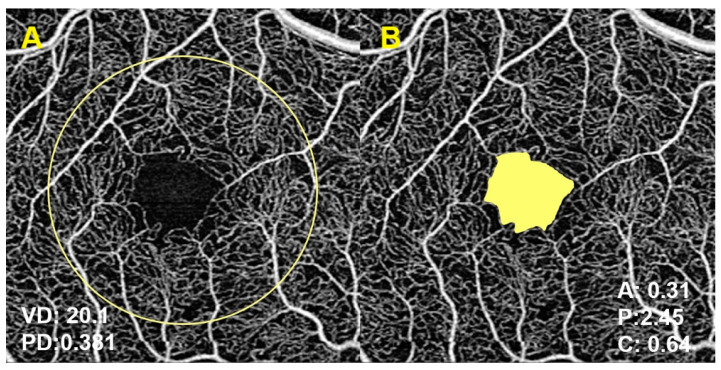
Optical coherence tomography angiography (OCTA) parameters evaluated in the study. (**A**): Vessel density (VD) and perfusion density (PD). (**B**): Foveal avascular zone parameters (A: area, P: perimeter, C: circularity). OCTA quantifications were performed by the device built-in commercial software (AngioPlex Metrix, Carl Zeiss Meditec, Dublin, CA, USA).

All OCTA images were captured in 3 × 3 mm cube scans using the same OCT device (Cirrus HD-OCT model 5000, Carl Zeiss Meditec, Dublin, CA, USA). OCTA quantifications were performed by the device built-in commercial software (AngioPlex Metrix, Carl Zeiss Meditec, Dublin, CA, USA) in the superficial capillary plexus (SCP). Measured variables included: vessel density (VD, mm^−1^), perfusion density (PD, ratio) and foveal avascular zone (FAZ) parameters: area (FAZa, mm^2^), perimeter (FAZp, mm) and circularity (FAZc, ratio) (Figure 1). OCTA image quality check was performed and scans with presence of artifacts, segmentation errors or signal strength index (SSI) < 7 were excluded from analysis.

### 2.5. Chronic Kidney Disease Stages and Risk Prognosis

**Figure 2:**
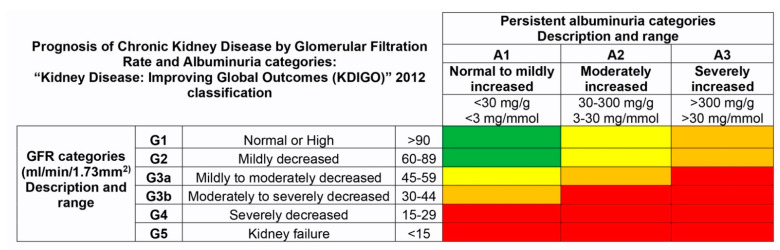
Prognosis of chronic kidney disease (CKD) by glomerular filtration rate (GFR) and albuminuria category, as per the “Kidney Disease: Improving Global Outcomes” (KDIGO) 2012 classification. Green, low risk (if no other markers of kidney disease, no CKD); yellow, moderately increased risk; orange, high risk; red, very high risk. (CKD, chronic kidney disease; GFR, glomerular filtration rate; KDIGO, Kidney Disease: Improving Global Outcomes). Table created using KDIGO CKD Workgroup Guidelines [3].

To assess diabetic renal damage, kidney function tests were performed and glomerular filtration rate (GFR) estimated with CKD-EPI [33] and urine albumin-to-creatinine ratio (ACR) were calculated. GFR and ACR groups were created according to the KDIGO (“Kidney Disease: Improving Global Outcomes”) 2012 Clinical Practice Guidelines classification (Figure 2) [3]. GFR was categorized as: G1, G2, G3a, G3b, G4, G5 (≥90, 60–89, 45–59, 30–44, 15–29 and <15 mL/min/1.73 m^2^ respectively). On the other hand, ACR was classified as: A1, A2 and A3 (<30, 30–300 and >300 mg/mg respectively). Finally, according to GFR and albuminuria categories, CKD risk probability was assigned under KDIGO Prognosis of CKD classification: low risk (G1 or G2 and A1), moderate risk (G1 or G2 and A2, G3a and A1), high risk (G1 or G2 and A3, G3a and A2, G3b and A1) and very high risk (G3a and A3, G3b and A2 or A3, G4 and A1, A2 or A3, G5 and A1, A2 or A3).

### 2.6. Statistical Analysis

Descriptive analysis was performed using frequencies and percentages were for qualitative variables and mean, standard deviation (SD), median and quartiles for quantitative variables. The Kolmogorov-Smirnov test was used to assess normality of distributions. For qualitative variables, the chi-squared test was used. For quantitative parametric variables group mean comparison, *p*-values from the generalized estimating equation (GEE) adjusted for age, gender, signal strength index (SSI), axial length, duration of DM disease and grade of DR were used. Bonferroni correction was used in multiple comparisons between group means. In nonparametric variables group mean comparisons, The Kruskal-Wallis test was used. To study the correlation between two continuous variables, the Spearman test was used. Also, receiver operating curves (ROC) were constructed to evaluate the area under the curve (AUC) of OCTA parameters. For all the tests, *p*-value < 0.05 was considered statistically significant. The statistical package R Studio (2.5) (R Studio, 02210 Boston, MA, USA) was used for the statistical analyses.

## 3. Results

### 3.1. Demographics and Baseline Characteristics of Study Cohort

**Figure 3:**
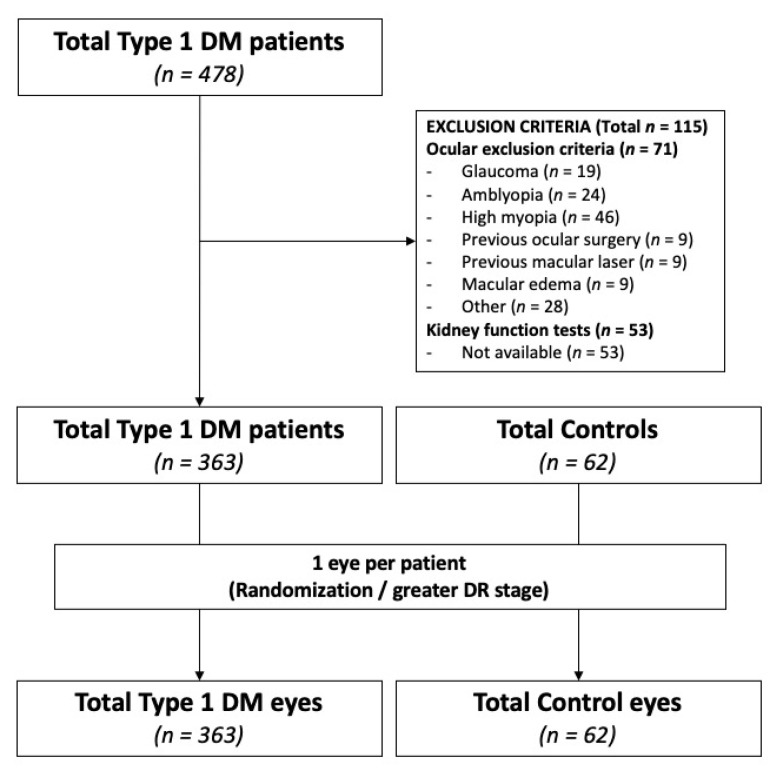
Consolidated standard of reporting trials (CONSORT)-style flow chart describing included and excluded patients and eyes in the study.

A consolidated standard of reporting trials (CONSORT)-style flow diagram describing included and excluded study eyes is presented in Figure 3.

A total number of 478 T1DM patients and 115 healthy controls underwent a complete ocular examination during the predetermined timeframe. Exclusion criteria were applied, and eyes were excluded due to ocular comorbidities (*n* = 71) or lack of kidney function tests (*n* = 53). To avoid risk of bilaterality bias, only one eye per patient was randomly selected, and in cases with asymmetric DR stage, the eye with higher DR grade was selected. A total number of 425 individuals, 363 T1DM patients and 62 healthy controls were finally included for statistical analysis. OCTA images with artifacts (*n* = 36), low quality (defined as SSI < 7, *n* = 71) or incorrect FAZ (*n* = 28) were excluded from analysis. Demographics, baseline characteristics including general characteristics, diabetes-related characteristics, such as DM duration, macrovascular complications, DR stage, systemic treatments and baseline bloods, of the study cohort disclosed by KDIGO prognosis categories are presented in Table 1.

### 3.2. OCTA Parameters and Kidney Function Tests

**Figure 4:**
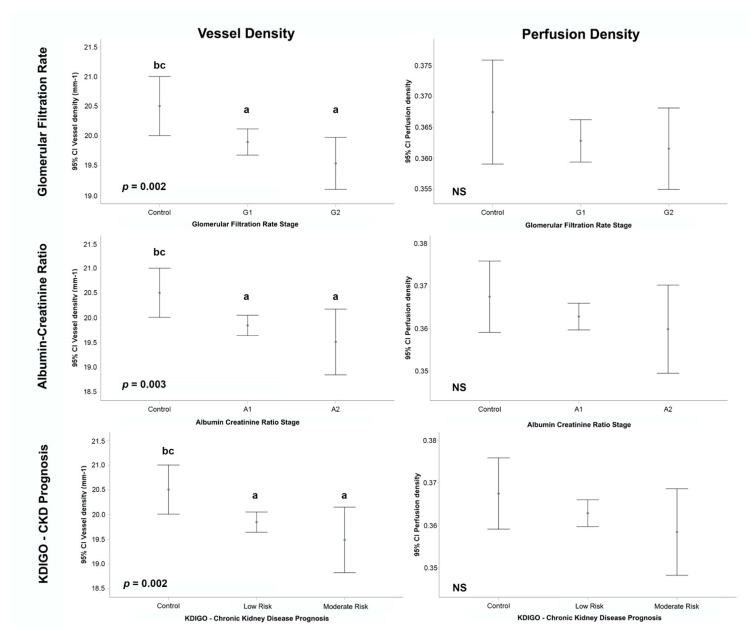
Vessel density, perfusion Density and kidney function tests, subgroup analysis by glomerular filtration rate (**top row**), albumin-creatinine ratio (**middle row**) and chronic kidney disease prognosis (**bottom row**) categories. *p*-values from the generalized estimating equation (GEE) adjusted by age, gender, signal strength index (SSI), axial length, duration of diabetes mellitus disease and grade of diabetic retinopathy. *p*-values for multiple comparisons adjusted using the Bonferroni method, 2 × 2 comparisons with *p* < 0.05: a vs. Control; b vs. G1/A1/Low risk; c vs. G2/A2/Moderate Risk. (CKD: chronic kidney disease, KDIGO: Kidney Disease: Improving Global Outcomes).

**Figure 5:**
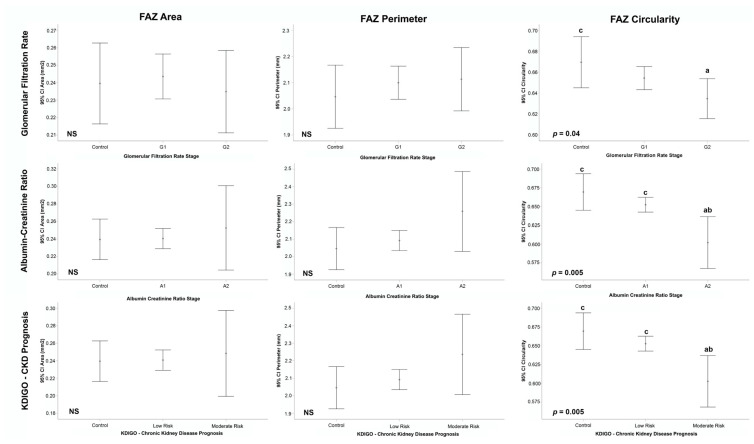
Foveal avascular zone area, perimeter and circularity and kidney function tests, subgroup analysis by glomerular filtration rate (**top row**), albumin-creatinine ratio (**middle row**) and chronic kidney disease prognosis (**bottom row**) categories. *p*-values from the generalized estimating equation (GEE) adjusted by age, gender, signal strength index (SSI), axial length, duration of diabetes mellitus disease and grade of diabetic retinopathy. *p*-values for multiple comparisons adjusted using the Bonferroni method. (2 × 2 comparisons with *p* < 0.05: a vs. Control; b vs. G1/A1/Low risk; c vs. G2/A2/Moderate Risk). (CKD: Chronic kidney disease, KDIGO: Kidney Disease: Improving Global Outcomes).

Quantifications of OCTA parameters were analyzed, and intergroup differences were calculated according to GFR, ACR and KDIGO prognosis categories, as summarized in Table 2 and Figure 4 and Figure 5. For statistical reasons, categories with very low numbers (*n* ≤ 3) were excluded from analysis (GFR G3a/G3b *n* = 3, ACR A3 *n* = 2, KDIGO high risk *n* = 3).

#### 3.2.1. Glomerular Filtration Rate

Mean GFR CKD-EPI calculated for T1DM cases was 100.68 ± 19.09 mL/min (mean ± SD) and 95.7 ± 22.98 mL/min for healthy controls. GFR categories included G1 (*n* = 263), G2 (*n* = 74) and G3a/G3b (*n* = 3). A significantly lower VD was observed in G1 and G2 categories compared to controls (19.9 ± 1.8 and 19.5 ± 1.9 vs. 20.5 ± 1.9, *p* = 0.002), overall as well as in 2 × 2 comparisons (*p* < 0.05 both). Similarly, lower FAZc was observed in G1 and G2 compared to controls (0.65 ± 0.1 and 0.63 ± 0.1 vs. 0.67 ± 0.1, *p* = 0.04), with significant differences between controls and G2 (*p* < 0.05). No differences were observed in PD, FAZa or FAZp.

#### 3.2.2. Albumin-Creatinine Ratio

Mean ACR was 9.96 ± 9.2 mg/g (mean ± SD) for T1DM and 9.5 ± 15.5 mg/g for healthy controls. ACR categories included A1 (*n* = 318), A2 (*n* = 20) and A3 (*n* = 2). Significant differences were observed between A1 and A2 categories and controls for VD (19.8 ± 1.9 and 19.5 ± 1.4 vs. 20.5 ± 1.9, *p* = 0.003), being both 2 × 2 comparisons significant (*p* < 0.05). Also, FAZc was significantly reduced in A1 and A2 categories compared to controls (0.65 ± 0.1 and 0.60 ± 0.1 vs. 0.67 ± 0.1, *p* = 0.005), with significant differences in 2 × 2 comparisons for A2 vs. controls (*p* < 0.05) and also for A2 vs. A1 (*p* < 0.05). A trend was observed for PD that did not reach significance level (*p* = 0.08), and no differences were observed in FAZa or FAZp.

#### 3.2.3. Kidney Disease: Improving Global Outcomes (KDIGO) Classification: Prognosis of Chronic Kidney Disease

T1DM patients were stratified according to the KDIGO 2012 CKD prognosis classification, based on the combination of the previous GFR and ACR categories. Two groups were identified as “Low” risk (G1 or G2 & A1, *n* = 317) or “Moderate” risk (G1 or G2 and A2, G3a and A1, *n* = 20). Three cases were found with greater stages (“High” risk, *n* = 3) and were excluded from this analysis.

VD was significantly reduced in patients with “Low” and “Moderate” risk categories compared to controls (19.8 ± 1.9 and 19.5 ± 1.4 vs. 20.5 ± 1.9, *p* = 0.002), with significant differences in both 2 × 2 comparisons. FAZc also showed significant differences and lower values were observed in “Low” and “Moderate” risk categories compared to controls (0.65 ± 0.1 and 0.60 ± 0.1 vs. 0.67 ± 0.1, *p* = 0.005). In 2 × 2 comparisons, significant differences were observed in FAZc between “Moderate” risk and controls (*p* < 0.05) and also “Low” risk patients (*p* < 0.05). PD showed a trend that was not significant (*p* = 0.06) and no differences were observed in FAZa or FAZp.

### 3.3. Correlations between Kidney Function Tests and OCTA Parameters: Influence of DM Duration and DR Grade

Correlation tests (Spearman’s rank correlation coefficient) were performed between OCTA parameters and GFR and ACR, overall and stratified by DM disease duration (<5 years, 5 to 15 years, and >15 years) and DR grades (no DR, non-proliferative DR and proliferative DR). For GFR, a non-significant positive trend was observed for FAZa in proliferative DR (*p* = 0.05), and no associations were observed for any other parameter, DR grade or DM duration groups. For ACR, a non-significant negative trend was observed for FAZc in T1DM with >15 years of DM duration (*p* = 0.08). No correlations were observed between ACR and any OCTA parameters, DM duration or DR grade.

### 3.4. Receiver Operating Curve (ROC) Analysis of OCTA Parameters and Chronic Kidney Disease Risk

**Figure 6:**
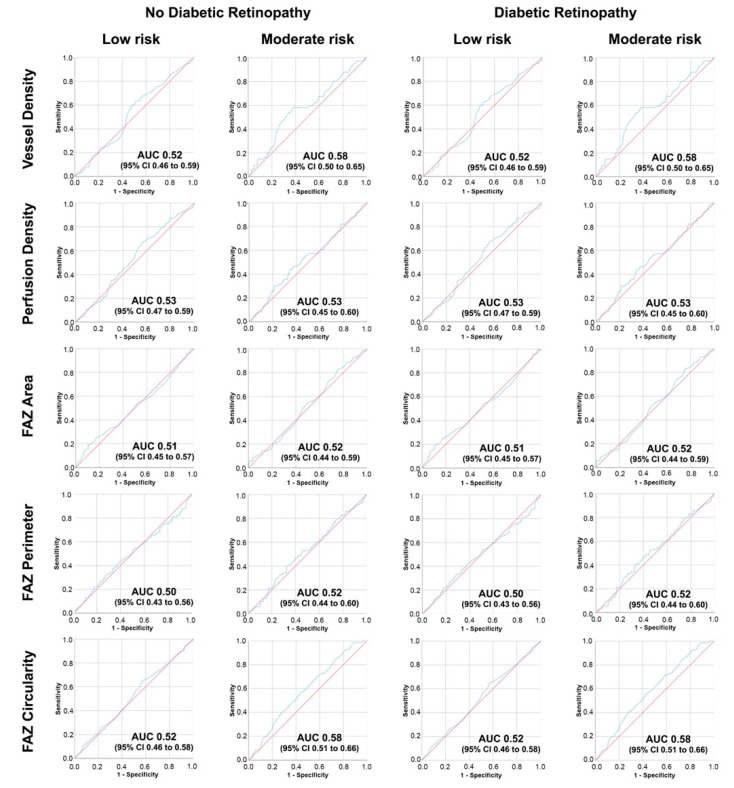
Receiver operating characteristic curve for each optical coherence tomography angiography (OCTA) parameter and “Kidney Disease: Improving Global Outcomes” KDIGO 2012 classification categories, as “low risk” (left column) and “moderate risk” (right column) in patients with no diabetic retinopathy (left two columns) and with diabetic retinopathy (right two columns).

Receiver operating curve (ROC) analysis were constructed to evaluate the diagnostic utility of OCTA parameters to detect patients with “low” and “moderate” risk of CKD according to KDIGO categories in non-DR and DR patients, presented in Figure 6. In T1DM patients with no DR, the best area under the curve (AUC) to identify patients with “moderate” risk was obtained for VD (AUC 0.58, 95% confidence interval, CI, 0.50 to 0.65) and FAZc (AUC 0.58, 95% CI 0.51 to 0.65). In T1DM DR patients, similar results were observed and VD (AUC 0.58, 95% CI 0.50 to 0.65) and FAZc (AUC 0.58, 95% CI 0.51 to 0.65) presented the highest AUC values.

## 4. Discussion

This report demonstrates the ability of specific OCTA parameters to identify different DKD categories in a non-invasive, objective quantitative way, using retinal images and blood test data from a large cohort of T1DM patients and controls collected prospectively. We describe that VD and FAZc parameters are able to detect different GFR, ACR and KDIGO categories in T1DM patients and controls, and we report that FAZc is able to discriminate within T1DM patients those with greater categories of DKD, and more importantly, those at greater risk of DKD progression. These findings highlight the potential of OCTA as a non-invasive tool to identify patients at risk of DKD progression in both specialized units and the community.

Since the advent of OCTA [34], several studies have been directed to investigate potential relationships between OCTA parameters and kidney function tests in DM, presenting controversial results (Table 3). We have observed a reduced VD in patients with greater categories of DKD in the three scales evaluated, GFR, ACR and KDIGO classifications. Consistently with these findings, some authors have reported associations between this parameter and GFR [7], with ACR [35] or with both tests [29,30]; however, other studies have not observed any relationship in DM patients with either no DR [13,16] or DR [20]. Nevertheless, there are considerable differences between these series and our cohort. First, almost all these previous series have been conducted in type 2 DM patients, the most prevalent type of DM, that commonly affects older patients and frequently presents other associated cardiovascular comorbidities, such as blood hypertension, dyslipidemia or metabolic syndrome. All these factors and others like the smoking status [36] could potentially have an influence on both kidney function and status of the retinal vascular network, affecting the results reported either way. Second, there are multiple technical differences in the OCT system (spectral domain vs. swept source), OCT device, scan size protocol (from 3 × 3 mm to 12 × 12 mm), capillary plexus evaluated (SCP vs. DCP) and software used (built-in vs. custom) for OCTA parameter quantification in each series, a relevant factor that adds to the variability of results and limits the inter-series comparison. Third, most of these series have been conducted in Asian populations, adding ethnicity as a potential confounder compared to our Caucasian cohort. Interestingly, the only study conducted in predominantly Caucasian population [28] (*n* = 10, 70% Caucasian, T2DM) did not found any association between VD and GFR, and only described an association with peripheral non-perfusion areas in wide field OCTA montages. Finally, some of these series present variable control of DM disease, as reflected in the wide range of HbA1c levels (6.7 to 9.4%) described. For all these reasons, the results reported in this study add to the existing evidence that VD and DKD appear to be associated in T1DM individuals.

One of the most clinically relevant findings of this study is the identification of FAZc as a potential indirect biomarker of DKD progression. The status of the FAZ and its different parameters, such as area, perimeter or circularity, has been previously described as a marker associated with DR stage [9,15,18], a surrogate marker of VA [40] or associated to HbA1c levels [24]. With regard to kidney function, some previous reports have described an association between FAZa and GFR [13] or ACR [27], although other authors have not found any association between these parameters [29]. In our series, we have not observed a significant association with FAZa or FAZp; nevertheless, FAZc has been proven effective to identify patients with greater DKD stages in the three scales evaluated, GFR, ACR and KDIGO prognosis classification. In particular, a reduced FAZc has been found in G2, A2 and moderate risk of CKD progression patients compared to controls, and more importantly, with A1 and low risk of CKD progression patients, respectively. This is an important feature, as it suggests that this parameter may be used as a non-invasive indirect biomarker of greater DKD stages in T1DM patients, and according to the KDIGO classification, greater risk of DKD progression. To present date, no previous reports in the literature have described possible relationships between FAZp or FAZc and kidney function tests. If confirmed in future series or longitudinal studies, this finding may highlight the potential role of OCTA as a diagnostic tool for DKD.

ROC curves have been constructed with the aim to evaluate the diagnostic potential of OCTA parameters for each category and DKD classification, as well as to investigate this performance separately in non-DR cases and cases with DR. In both subgroups, the greatest AUC values were observed for VD and FAZc to identify cases with greater categories of ACR (A2) and KDIGO (Moderate risk) classifications. These results suggest that OCTA may be useful to detect subgroups of patients with kidney disease, or even more importantly, identify patients at risk of DKD progression in a non-invasive way. The implications of this finding are relevant as a blood test is currently required to assess this risk. In this area of research, recent studies have highlighted the potential of artificial intelligence (AI) algorithms to identify CKD categories from retinal photographs using existing datasets from DR screening programs [41,42]. However, the performance of these models is not directly comparable to our results for a variety of reasons. The training of these AI algorithms requires large volumes of images, mostly include only type 2 DM cases and are dependent on the type of fundus camera evaluated, often with poor external validity [43]. Conversely, our quantitative method is deployable as it involves the use of a standard OCT device with the built-in quantification software available in its commercial version. Although at the present time both approaches need to be independently validated, their combination appears extremely interesting and the application of AI algorithms to the rich granular data of OCTA images and OCTA-derived quantifications will be investigated in the next future.

This study has a number of strengths and limitations. The large study cohort, recruited prospectively with collection of high-quality ocular and systemic data, the strict exclusion criteria applied to cases and OCTA images, the selection of 1 eye per patient to avoid risk of bilaterality bias and the presence of a control group are the main strengths of this study. Limitations include the use of a commercial software that allows the evaluation of only the SCP and not the DCP and the lack of T1DM patients with advanced categories of CKD.

## 5. Conclusions

In conclusion, this study demonstrates that specific OCTA parameters, such as VD and FAZc, are able to differentiate distinct DKD categories in a non-invasive, objective and quantitative way in T1DM patients and controls. Moreover, we specifically describe that FAZc could considered a biomarker of DKD progression in T1DM individuals, being able to identify patients at greater risk according to the KDIGO prognosis classification. If these findings are confirmed in future studies, OCTA may play a relevant role in the management of DKD in different scenarios, such as highly specialized Diabetes units and, potentially, deployed in a community setting, as a non-invasive helpful tool to identify patients with greater categories of DKD or higher risk of DKD progression.

## Figures and Tables

**Table 1:** Demographics and baseline characteristics of study patients according to KDIGO Prognosis categories.

			Prognosis of CKD (Risk)			
Variable	Statistic	Control (*n* = 62)	Low (*n* = 337)	Moderate (*n* = 23)	High (*n* = 3)	*p*-Value *
**General characteristics**						
Age (years)	Mean (SD)	44.6 (13.5)	39 (11.4)	44.7 (15.8)	58.5 (6.6)	<0.001
	Median (IQR)	46.5 (33; 56.8)	37.4 (30.2; 47.4)	46 (30.3; 55.4)	62 (50.9; 62.6)	
Sex (female)	*n* (%)	43 (69.4%)	173 (51.3%)	10 (43.5%)	1 (33.3%)	0.110
Smoking habits						<0.001
- Non-smoker	*n* (%)	45 (72.6%)	208 (61.9%)	14 (60.9%)	0 (0%)	
- Actual smoker	*n* (%)	4 (6.5%)	73 (21.7%)	6 (26.1%)	0 (0%)	
- Ex-smoker	*n* (%)	13 (21%)	55 (16.4%)	3 (13%)	3 (100%)	
Hypertension	*n* (%)	0 (0%)	25 (7.4%)	10 (43.5%)	3 (100%)	<0.001
BMI (kg/m^2^)	Mean (SD)	23.2 (3.4)	24.8 (3.6)	25.1 (4.8)	26.8 (5.8)	0.091
	Median (IQR)	23 (20.8; 25.3)	24.2 (22.3; 27.1)	24 (21.6; 27.9)	24.6 (22.3; 33.3)	
**DM-related features**						
DM duration (years)	Mean (SD)	0 (0)	19.5 (10.5)	26.9 (14.1)	30.3 (14.9)	0.017
	Median (IQR)	0 (0; 0)	19.4 (10.5; 26.7)	30.6 (15.4; 37.7)	37.8 (13.1; 40)	
Macrovascular complications						
- Cerebrovascular disease	*n* (%)	0 (0%)	4 (1.2%)	0 (0%)	0 (0%)	0.762
- Ischemic heart disease	*n* (%)	0 (0%)	3 (0.9%)	0 (0%)	1 (50%)	<0.001
- Peripheral vasculopathy	*n* (%)	0 (0%)	1 (0.3%)	0 (0%)	1 (50%)	<0.001
Diabetic Retinopathy Stage						<0.001
- No retinopathy	*n* (%)	-	229 (68.2%)	13 (56.5%)	1 (33.3%)	
- NP Mild	*n* (%)	-	86 (25.6%)	6 (26.1%)	0 (0%)	
- NP Moderate	*n* (%)	-	16 (4.8%)	1 (4.3%)	0 (0%)	
- NP Severe	*n* (%)	-	1 (0.3%)	0 (0%)	2 (66.7%)	
- Proliferative	*n* (%)	-	4 (1.2%)	3 (13%)	0 (0%)	
**Systemic Treatment**						
- Insulin requirements (IU/kg/day)	Mean (SD)	-	0.6 (0.2)	0.7 (0.3)	0.6 (0.2)	<0.001
	Median (IQR)		0.6 (0.5; 0.8)	0.6 (0.5; 0.9)	0.7 (0.4; 0.7)	
- Insulin pump	*n* (%)	-	61 (18.1%)	4 (17.4%)	0 (0%)	0.716
- ACEI or ARB treatment	*n* (%)	0 (0%)	30 (8.9%)	11 (47.8%)	2 (66.7%)	<0.001
- Statins treatment	*n* (%)	0 (0%)	63 (18.7%)	7 (30.4%)	2 (66.7%)	0.002
- Antiplatelet treatment	*n* (%)	0 (0%)	13 (3.9%)	1 (4.3%)	1 (33.3%)	0.013
**Laboratory tests**						
HbA1c (%)	Mean (SD)	5.3 (0.3)	7.5 (1)	7.5 (0.7)	7.9 (0.9)	<0.001
	Median (IQR)	5.3 (5.1; 5.6)	7.4 (6.8; 7.9)	7.6 (7.1; 7.7)	7.6 (7.2; 8.9)	
Total cholesterol (mg/dL)	Mean (SD)	193.8 (33.1)	176.9 (31)	184 (29.6)	171 (32)	<0.001
	Median (IQR)	192 (169; 216)	175 (156; 195)	180 (170; 206)	189 (134; 190)	
LDL cholesterol (mg/dL)	Mean (SD)	115.6 (32)	101.9 (24.4)	106.1 (22.1)	89 (26)	0.005
	Median (IQR)	113.5 (92; 144)	101 (84.5; 119)	102.5 (90; 125)	103 (59; 105)	
HDL cholesterol (mg/dL)	Mean (SD)	57.1 (14.1)	59.5 (17.4)	59 (15.2)	63.3 (9.3)	0.787
	Median (IQR)	56.5 (48; 67)	56 (47; 69)	58 (49; 70)	59 (57; 74)	
Triglycerides (md/dL)	Mean (SD)	109.8 (58.5)	80.9 (50.6)	96.5 (52.9)	94 (28.6)	<0.001
	Median (IQR)	98.5 (66; 144)	67 (53; 90)	76 (56; 128)	91 (67; 124)	
Hemoglobin (g/L)	Mean (SD)	135.1 (11.3)	141.8 (12.6)	143.5 (12.5)	129.7 (20.8)	0.009
	Median (IQR)	134 (128; 140)	141 (133; 151)	145 (134; 151)	138 (106; 145)	
Platetets (10^9^/L)	Mean (SD)	251.6 (53.9)	252.4 (57)	255.7 (74.7)	281 (98.5)	0.857
	Median (IQR)	244.5 (207; 296)	249 (212; 290)	244 (204; 272)	326 (168; 349)	
ACR (mg/g)	Mean (SD)	9.5 (15.5)	4.9 (5)	68.8 (51.4)	126.7 (157.4)	<0.001
	Median (IQR)	3 (2; 8)	3 (2; 6)	48 (33; 110)	68 (7; 305)	

DM: Diabetes mellitus; OCTA: optical coherence tomography angiography; ACEI: angiotensin-converting enzyme inhibitors; ARB: angiotensin II receptor blocker; HbA1c: glycated hemoglobin (A1c); BMI: body mass index; DM: diabetes mellitus; IU: insulin units LDL-c: low-density lipoprotein-cholesterol; HDL-c: high-density lipoprotein-cholesterol; Hb: hemoglobin; ACR: albumin-to-creatinine ratio; SD: standard deviation; IQR: interquartile range. * *p*-values for frequencies from the chi-squared test and for continuous variables from the Kruskal-Wallis and ANOVA tests.

**Table 2:** Optical coherence tomography angiography (OCTA) measurements according to kidney function test classifications and categories.

			GFR Category			ACR Category			Prognosis of CKD (Risk)		
Variable	Statistic	Control	G1	G2	*p*-Value *	A1	A2	*p*-Value *	Low	Moderate	*p*-Value *
**Vessel density**	Mean (SD)	20.5 (1.9)	19.9 (1.8)	19.5 (1.9)	0.002 ^ab^	19.8 (1.9)	19.5 (1.4)	0.003 ^cd^	19.8 (1.9)	19.5 (1.4)	0.002 ^f,g^
**(mm^−1^)**	Median (IQR)	21 (20; 21.8)	20.1 (18.8; 21.2)	19.4 (18.6; 21.1)		20.1 (18.8; 21.2)	19.9 (18.6; 20.4)		20.1 (18.8; 21.2)	19.9 (18.6; 20.4)	
	*n*	59	263	74		318	20		317	20	
**Perfusion density**	Mean (SD)	0.37 (0.03)	0.36 (0.03)	0.36 (0.03)	0.111	0.36 (0.03)	0.36 (0.02)	0.084	0.36 (0.03)	0.36 (0.02)	0.062
**(0–1)**	Median (IQR)	0.38 (0.36; 0.39)	0.37 (0.35; 0.38)	0.37 (0.34; 0.38)		0.37 (0.35; 0.38)	0.36 (0.35; 0.38)		0.37 (0.35; 0.38)	0.36 (0.35; 0.38)	
	*n*	59	263	73		317	20		316	20	
**FAZ area**	Mean (SD)	0.24 (0.08)	0.24 (0.1)	0.23 (0.1)	0.919	0.24 (0.1)	0.25 (0.1)	0.890	0.24 (0.1)	0.25 (0.1)	0.972
**(mm^2^)**	Median (IQR)	0.24 (0.19; 0.29)	0.23 (0.17; 0.31)	0.23 (0.18; 0.29)		0.23 (0.17; 0.3)	0.25 (0.17; 0.33)		0.23 (0.17; 0.3)	0.23 (0.17; 0.33)	
	*n*	53	244	70		296	19		295	19	
**FAZ perimeter**	Mean (SD)	2.05 (0.44)	2.1 (0.5)	2.11 (0.51)	0.714	2.09 (0.5)	2.26 (0.47)	0.309	2.09 (0.5)	2.24 (0.48)	0.442
**(mm)**	Median (IQR)	2.09 (1.79; 2.29)	2.1 (1.77; 2.44)	2.15 (1.83; 2.44)		2.11 (1.78; 2.43)	2.38 (1.95; 2.59)		2.11 (1.77; 2.43)	2.09 (1.95; 2.59)	
	*n*	53	244	70		296	19		295	19	
**FAZ circularity**	Mean (SD)	0.67 (0.09)	0.65 (0.09)	0.63 (0.08)	0.040 ^b^	0.65 (0.09)	0.60 (0.07)	0.005 ^de^	0.65 (0.09) ^h^	0.60 (0.07) ^h^	0.005 ^f^
**(0–1)**	Median (IQR)	0.69 (0.62; 0.73)	0.67 (0.6; 0.72)	0.65 (0.58; 0.7)		0.67 (0.6; 0.71)	0.61 (0.55; 0.65)		0.67 (0.6; 0.71)	0.61 (0.55; 0.65)	
	*n*	53	244	70		296	19		295	19	

OCTA: optical coherence tomography angiography; GFR: glomerular filtration rate; ACR: albumin-to-creatinine ratio; FAZ: foveal avascular zone; SD: standard deviation; IQR: interquartile range. * *p*-values from the Kruskal-Wallis test. *p*-values for multiple comparisons adjusted using the Bonferroni method. (2 × 2 Intergroup differences with *p* < 0.05: ^a^ Control vs. G1; ^b^ Control vs. G2; ^c^ Control vs. A1; ^d^ Control vs. A2; ^e^ A1 vs. A2; ^f^ Control vs. Low; ^g^ Control vs. Moderate; ^h^ Low vs. Moderate).

**Table 3:** Selection of relevant papers published to date on OCTA and diabetic kidney disease.

Author	Year	DM Patients	Controls	Diabetic Retinopathy Grades (%)	Association between OCTA and Kidney Function Parameters	Conclusions
Tang et al. [20]	2017	286	-	39.4/27.6/26.3/6.7 *	no	No association between OCTA (VD, FAZa, FAZc, FD, VDI) and GFR
Ting et al. [7]	2017	50	-	19/17/21/22/21	yes	Association between capillary density index and GFR
Cao et al. [16]	2018	71	67	No DR	no	No association between VD and serum creatinine
Lee et al. [13]	2018	74	34	No DR	yes	Association between FAZa and eGFR
Ahmadzadeh-Amiri et al. [27]	2019	46	57	NPDR 60.3/PDR 39.7	yes	Association between FAZa and ACR.
Cankurtaran et al. [35]	2020	86	51	No DR	yes	Association between VD and ACR
Tom et al. [28]	2020	10	-	No DR 60/any DR 40	yes	Association between GFR and retinal non perfusion
Wang et al. [30]	2020	874	-	No DR 87.9/any DR 12.1	yes	Association between VD and GFR/VD and MAU
Zhuang et al. [29]	2020	150	-	No DR 24.7/ NPDR 59.3/PDR 16	yes	association between: VD and GFR/VD and ACR
Shaw et al. [37]	2021	52	-	No DR	no	No association between OCTA (VD, FAZa) and GFR or ACR
Ucgul Atilgan et al. [38]	2021	70	-	No DR 57.14/mild 42.86	yes	Association between: VD and MAU/VD and creatinine
Oliveira da Silva et al. [39]	2021	65	37	No DR 81.39/mild 20.93	yes	Association between: FAZa and DKD/VD and DKD
***This study***	***2021***	***478***	***115***	***64.1/26.1/4.5/0.6/4.6***	***yes***	***Decreased VD and FAZc in greater GFR, ACR and KDIGO grades***

Selection of relevant papers published to date on OCTA and diabetic kidney disease. In bold and italic, details of the present study. OCTA: optical coherence tomography angiography; diabetic retinopathy (DR) grades: absent/mild/moderate/severe/proliferative; * diabetic retinopathy (DR) grades modified groups: absent/mild/moderate/severe or worse DR; NPDR: non-proliferative diabetic retinopathy; PDR: proliferative diabetic retinopathy; GFR: glomerular filtration rate; MAU: microalbuminuria; AER: albumin excretion rate in 24 h; ACR: albumin-to-creatinine ratio; VD: vessel density; VDI: vessel diameter index; PD: perfusion density; FD: fractal dimension; FAZa: foveal avascular zone area; FAZp: foveal avascular zone perimeter; FAZc: foveal avascular zone circularity; SCP: superficial capillary plexus, DCP: deep capillary plexus; KDIGO: Kidney Disease: Improving Global Outcomes.

## Data Availability

The datasets used and/or analyzed during the current study are available from the corresponding author on reasonable request.

## References

[1] Faselis C., Katsimardou A., Imprialos K., Deligkaris P., Kallistratos M., Dimitriadis K. (2019). Microvascular Complications of Type 2 Diabetes Mellitus. Curr. Vasc. Pharmacol..

[2] Bourne R.R.A., Stevens G.A., White R.A., Smith J.L., Flaxman S.R., Price H., Jonas J.B., Keeffe J., Leasher J., Naidoo K. (2013). Causes of vision loss worldwide, 1990–2010: A systematic analysis. Lancet Glob. Health.

[3] Kidney Disease: Improving Global Outcomes (KDIGO), CKD Work Group (2013). KDIGO clinical practice guideline for the evaluation and management of chronic kidney disease. Kidney Int. Suppl..

[4] Kramer C.K., Retnakaran R. (2013). Concordance of retinopathy and nephropathy over time in Type 1 diabetes: An analysis of data from the Diabetes Control and Complications Trial. Diabet. Med..

[5] Bjerg L., Hulman A., Charles M., Jørgensen M.E., Witte D.R. (2018). Clustering of microvascular complications in Type 1 diabetes mellitus. J. Diabetes Complic..

[6] Jia Y., Tan O., Tokayer J., Potsaid B., Wang Y., Jonathan J., Kraus M.F., Subhash H., Fujimoto J.G., Hornegger J. (2012). Angiography With Optical Coherence Tomography. Clin. Sci..

[7] Ting D.S.W., Tan G.S.W., Agrawal R., Yanagi Y., Sie N.M., Wong C.W., Yeo I.Y.S., Lee S.Y., Cheung C.M.G., Wong T.Y. (2017). Optical coherence tomographic angiography in type 2 diabetes and diabetic retinopathy. JAMA Ophthalmol..

[8] Kalra G., Zarranz-Ventura J., Chahal R., Bernal-Morales C., Lupidi M., Chhablani J. (2021). Optical computed tomography (OCT) angiolytics: A review of OCT angiography quantiative biomarkers. Surv. Ophthalmol..

[9] Dimitrova G., Chihara E., Takahashi H., Amano H., Okazaki K. (2017). Quantitative retinal optical coherence tomography angiography in patients with diabetes without diabetic retinopathy. Investig. Ophthalmol. Vis. Sci..

[10] Ishibazawa A., Nagaoka T., Takahashi A., Omae T., Tani T., Sogawa K., Yokota H., Yoshida A. (2015). Optical coherence tomography angiography in diabetic retinopathy: A prospective pilot study. Am. J. Ophthalmol..

[11] Agemy S.A., Scripsema N.K., Shah C.M., Chui T., Garcia P.M., Lee J.G., Gentile R.C., Hsiao Y.S., Zhou Q., Ko T. (2015). Retinal vascular perfusion density mapping using optical coherence tomography angiography in normals and diabetic retinopathy patients. Retina.

[12] Nesper P.L., Roberts P.K., Onishi A.C., Chai H., Liu L., Jampol L.M., Fawzi A.A. (2017). Quantifying Microvascular Abnormalities With Increasing Severity of Diabetic Retinopathy Using Optical Coherence Tomography Angiography. Investig. Ophthalmol. Vis. Sci..

[13] Lee D.H., Yi H.C., Bae S.H., Cho J.H., Choi S.W., Kim H. (2018). Risk factors for retinal microvascular impairment in type 2 diabetic patients without diabetic retinopathy. PLoS ONE.

[14] Kim A.Y., Chu Z., Shahidzadeh A., Wang R.K., Puliafito C.A., Kashani A.H. (2016). Quantifying microvascular density and morphology in diabetic retinopathy using spectral-domain optical coherence tomography angiography. Investig. Ophthalmol. Vis. Sci..

[15] Barraso M., Alé-Chilet A., Hernández T., Oliva C., Vinagre I., Ortega E., Figueras-Roca M., Sala-Puigdollers A., Esquinas C., Esmatjes E. (2020). Optical Coherence Tomography Angiography in Type 1 Diabetes Mellitus. Report 1: Diabetic Retinopathy. Transl. Vis. Sci. Technol..

[16] Cao D., Yang D., Huang Z., Zeng Y., Wang J., Hu Y., Zhang L. (2018). Optical coherence tomography angiography discerns preclinical diabetic retinopathy in eyes of patients with type 2 diabetes without clinical diabetic retinopathy. Acta Diabetol..

[17] Kim M., Choi S.Y., Park Y.-H. (2018). Quantitative analysis of retinal and choroidal microvascular changes in patients with diabetes. Sci. Rep..

[18] De Carlo T.E., Chin A.T., Bonini Filho M.A., Adhi M., Branchini L., Salz D.A., Baumal C.R., Crawford C., Reichel E., Witkin A.J. (2015). Detection of microvascular changes in eyes of patients with diabetes but not clinical diabetic retinopathy using optical coherence tomography angiography. Retina.

[19] Forte R., Haulani H., Jürgens I. (2020). Quantitative and qualitative analysis of the three capillary plexuses and choriocapillaris in patients with type 1 and type 2 diabetes mellitus without clinical signs of diabetic retinopathy. A prospective pilot study. Retina.

[20] Tang F.Y., Ng D.S., Lam A., Luk F., Wong R., Chan C., Mohamed S., Fong A., Lok J., Tso T. (2017). Determinants of Quantitative Optical Coherence Tomography Angiography Metrics in Patients with Diabetes. Sci. Rep..

[21] Liu G., Xu D., Wang F. (2018). New insights into diabetic retinopathy by OCT angiography. Diabetes Res. Clin. Pract..

[22] Khadamy J., Aghdam K.A., Falavarjani K.G. (2018). An Update on Optical Coherence Tomography Angiography in Diabetic Retinopathy. J. Ophthalmic Vis. Res..

[23] Lu Y., Simonett J.M., Wang J., Zhang M., Hwang T., Hagag A.M., Huang D., Li D., Jia Y. (2018). Evaluation of automatically quantified foveal avascular zone metrics for diagnosis of diabetic retinopathy using optical coherence tomography angiography. Investig. Ophthalmol. Vis. Sci..

[24] Bernal-Morales C., Alé-Chilet A., Martín-Pinardel R., Barraso M., Hernández T., Oliva C., Vinagre I., Ortega E., Figueras-Roca M., Sala-Puigdollers A. (2021). Optical Coherence Tomography Angiography in Type 1 Diabetes Mellitus. Report 4: Glycated Haemoglobin. Diagnostics.

[25] Vadalà M., Castellucci M., Guarrasi G., Terrasi M., La Blasca T., Mulè G. (2019). Retinal and choroidal vasculature changes associated with chronic kidney disease. Graefe’s Arch. Clin. Exp. Ophthalmol..

[26] Yeung L., Wu I., Sun C., Liu C., Chen S., Tseng C., Lee H., Lee C. (2019). Early retinal microvascular abnormalities in patients with chronic kidney disease. Microcirculation.

[27] Ahmadzadeh Amiri A., Sheikh Rezaee M.R., Ahmadzadeh Amiri A., Soleymanian T., Jafari R., Ahmadzadeh Amiri A. (2020). Macular Optical Coherence Tomography Angiography in Nephropathic Patients with Diabetic Retinopathy in Iran: A Prospective Case–Control Study. Ophthalmol. Ther..

[28] Tom E.S., Saraf S.S., Wang F., Zhang Q., Vangipuram G., Limonte C.P., de Boer I.H., Wang R.K., Rezaei K.A. (2020). Retinal Capillary Nonperfusion on OCT-Angiography and Its Relationship to Kidney Function in Patients with Diabetes. J. Ophthalmol..

[29] Zhuang X., Cao D., Zeng Y., Yang D., Yao J., Kuang J., Xie J., He M., Cai D., Zhang S. (2020). Associations between retinal microvasculature/microstructure and renal function in type 2 diabetes patients with early chronic kidney disease. Diabetes Res. Clin. Pract..

[30] Wang W., He M., Gong X., Wang L., Meng J., Li Y., Xiong K., Li W., Huang W. (2020). Association of renal function with retinal vessel density in patients with type 2 diabetes by using swept-source optical coherence tomographic angiography. Br. J. Ophthalmol..

[31] Zarranz-Ventura J., Barraso M., Alé-Chilet A., Hernandez T., Oliva C., Gascón J., Sala-Puigdollers A., Figueras-Roca M., Vinagre I., Ortega E. (2019). Evaluation of microvascular changes in the perifoveal vascular network using optical coherence tomography angiography (OCTA) in type I diabetes mellitus: A large scale prospective trial. BMC Med. Imaging.

[32] Wilkinson C.P., Ferris F.L., Klein R.E., Lee P.P., Agardh C.D., Davis M., Dills D., Kampik A., Pararajasegaram R., Verdaguer J.T. (2003). Proposed international clinical diabetic retinopathy and diabetic macular edema disease severity scales. Ophthalmology.

[33] Levey A.S., Stevens L.A., Schmid C.H., Zhang Y., Castro A.F., Feldman H.I., Kusek J.W., Eggers P., Van Lente F., Greene T. (2009). A New Equation to Estimate Glomerular Filtration Rate. Ann. Intern. Med..

[34] Spaide R.F., Fujimoto J.G., Waheed N.K., Sadda S.R., Staurenghi G., States U., Science C., States U., Angeles L., States U. (2018). Optical coherence tomography angiography. Prog. Retin. Eye Res..

[35] Cankurtaran V., Inanc M., Tekin K., Turgut F. (2020). Retinal Microcirculation in Predicting Diabetic Nephropathy in Type 2 Diabetic Patients without Retinopathy. Ophthalmologica.

[36] Liu D.W., Haq Z., Yang D., Stewart J.M. (2021). Association between smoking history and optical coherence tomography angiography findings in diabetic patients without diabetic retinopathy. PLoS ONE.

[37] Shaw L.T., Khanna S., Chun L.Y., Dimitroyannis R.C., Rodriguez S.H., Massamba N., Hariprasad S.M., Skondra D. (2021). Quantitative optical coherence tomography angiography (Octa) parameters in a black diabetic population and correlations with systemic diseases. Cells.

[38] Ucgul Atilgan C., Atilgan K.G., Kosekahya P., Goker Y.S., Karatepe M.S., Caglayan M., Citirik M. (2021). Retinal Microcirculation Alterations in Microalbuminuric Diabetic Patients with and without Retinopathy. Semin. Ophthalmol..

[39] da Silva M.O., do Carmo Chaves A.E.C., Gobbato G.C., dos Reis M.A., Lavinsky F., Schaan B.D., Lavinsky D. (2021). Early neurovascular retinal changes detected by swept-source OCT in type 2 diabetes and association with diabetic kidney disease. Int. J. Retin. Vitr..

[40] Kim K., Kim E.S., Yu S.-Y. (2018). Optical coherence tomography angiography analysis of foveal microvascular changes and inner retinal layer thinning in patients with diabetes. Br. J. Ophthalmol..

[41] Sabanayagam C., Xu D., Ting D.S.W., Nusinovici S., Banu R., Hamzah H., Lim C., Tham Y., Cheung C.Y., Tai E.S. (2020). Articles A deep learning algorithm to detect chronic kidney disease from retinal photographs in community-based populations. Lancet.

[42] Zhang K., Liu X., Xu J., Yuan J., Cai W., Chen T., Wang K., Gao Y., Nie S., Xu X. (2021). Deep-learning models for the detection and incidence prediction of chronic kidney disease and type 2 diabetes from retinal fundus images. Nat. Biomed. Eng..

[43] Lee A.Y., Yanagihara R.T., Lee C.S., Blazes M., Jung H.C., Chee Y.E., Gencarella M.D., Gee H., Maa A.Y., Cockerham G.C. (2021). Multicenter, Head-to-Head, Real-World Validation Study of Seven Automated Artificial Intelligence Diabetic Retinopathy Screening Systems. Diabetes Care.

